# Analysis of the Magnetic Field Influence on the Rheological Properties of Healthy Persons Blood

**DOI:** 10.1155/2013/490410

**Published:** 2013-09-02

**Authors:** Anna Marcinkowska-Gapinska, Honorata Nawrocka-Bogusz

**Affiliations:** ^1^Rheological Laboratory, Department of Neurology, Karol Marcinkowski University of Medical Sciences in Poznań, Przybyszewskiego 49, 60-355 Poznań, Poland; ^2^Department of Biophysics, Poznań University of Medical Sciences, Fredry 10, 61-701 Poznań, Poland

## Abstract

The influence of magnetic field on whole blood rheological properties remains a weakly known phenomenon. An *in vitro* analysis of the magnetic field influence on the rheological properties of healthy persons blood is presented in this work. The study was performed on blood samples taken from 25 healthy nonsmoking persons and included comparative analysis of the results of both the standard rotary method (flow curve measurement) and the oscillatory method known also as the mechanical dynamic analysis, performed before and after exposition of blood samples to magnetic field. The principle of the oscillatory technique lies in determining the amplitude and phase of the oscillations of the studied sample subjected to action of a harmonic force of controlled amplitude and frequency. The flow curve measurement involved determining the shear rate dependence of blood viscosity. The viscoelastic properties of the blood samples were analyzed in terms of complex blood viscosity. All the measurements have been performed by means of the Contraves LS40 rheometer. The data obtained from the flow curve measurements complemented by hematocrit and plasma viscosity measurements have been analyzed using the rheological model of Quemada. No significant changes of the studied rheological parameters have been found.

## 1. Introduction

Artificial electromagnetic fields disturb the geomagnetic field and influence human organism causing different symptoms: headache, hyperactivity, fatigue, emotional tension, daily rhythm disturbances, and so forth. Fast changing magnetic fields are considered harmful, whereas weak and slow changing magnetic fields (MFs) are used in the diagnosis and in the treatment of many diseases. The use of variable magnetic fields in medicine covers many areas such as orthopedics, rheumatology, internal medicine, neurology, psychiatry, dentistry, and also psychiatry [1, 2].

The biophysical mechanisms of the action of variable low frequency magnetic fields are the influence on uncompensated magnetic spins of paramagnetic elements, free oxygen radicals, and diamagnetic molecules. Variable magnetic fields act on components of cell membranes having the properties of liquid crystal. They influence the depolarization of cells by introducing an additional force which changes positions of moving electric charges and induce potential in the areas filled with electrolyte. Variable magnetic fields not only cause the intensification of the process of the oxygen utilization and the tissue respiration increase reparation processes and the regeneration of soft tissues but also show a hypoglycemic effect and cause the acceleration of bone healing [1].

The effect of magnetic field on the rheological properties of blood is not well known yet. One of the effects observed so far is the decrease in the whole blood viscosity [3]. It has also been observed that the Viofor JPS device caused improvement in upper limb blood flow immediately after the first treatment. The measurements were performed before and after MF application; the results were observed by means of thermography [4]. 

It is believed that there is a direct relation between the rheological properties of systemic fluids and the processes taking place in living organisms [5]. The flow of blood through blood vessels is a very complex phenomenon due to physical and physicochemical properties of blood and the structure of the circulatory system. Features related to the flow of blood in blood vessels are referred to as blood fluidity [6]. Rheological characteristics of every material depend mainly on two parameters: viscosity and elasticity. Viscosity is a parameter determining the resistance of the material to flow, while elasticity expresses the material resistance against deformation. A hemorheological study is primarily based on the blood viscosity measurements. In the case of non-Newtonian fluids such as blood, viscosity is a function of applied shear rate. The main factors determining blood viscosity are hematocrit value, erythrocytes aggregability and deformability, and the plasma viscosity [7].

Measurements of whole blood viscosity as a function of shear rate are performed by means of rotary rheometers. On the contrary, blood plasma viscosity can be measured both by rotary and capillary viscometers because it is a Newtonian liquid [7–9]. Additional information about blood rheology can be achieved from nonviscometric oscillatory measurements, called also dynamic mechanical analysis (DMA). The principle of the oscillatory technique lies in determining the amplitude and phase of the oscillations of the studied sample subjected to action of a harmonic force of controlled amplitude and frequency. A measurement performed by means of the oscillatory method should provide information about viscoelastic properties of the fluid under study which can be expressed in terms of a complex viscosity:

(1) $$\begin{array}{c} \eta^{*}=\eta^{\prime }+i\eta^{\prime \prime }, \end{array}$$

where η* is the complex viscosity, *η*′ is the viscous component, and *η*′′ is the elastic component.

Additional information about erythrocytes properties, similar to that from direct measurements of red cells aggregability and deformability [7, 8, 10, 11], can be achieved indirectly from a mathematical analysis of the flow curve with the use of rheological models [7, 12]. In this work we use the Quemada model [13, 14]. 

The aim of the present work was a comprehensive analysis of the rheological properties of blood in a group of healthy objects with the use of a rotary-oscillating rheometer. Both rotary and oscillatory measurements were performed in this study. We present the results of viscosity measurements of both whole blood and blood plasma (rotary method) and the results of complex viscosity measurements with separately calculated viscous and elastic components of the blood complex viscosity. The flow curves were analyzed with the help of Quemada rheological model to estimate the parameters measuring red cells aggregability and deformability in healthy patients before and after application of magnetic field.

## 2. Material and Methods

Blood samples for the comprehensive rheological analysis were taken from a group of 25 healthy nonsmoking patients, aged 21 to 51, with 19 men and 6 women. The average age of patients in the group was 31. Blood donors did not suffer from any chronic diseases and passed the standard tests for blood donors. The interval between the blood collection and the viscosity measurement never exceeded 4 hours. The comprehensive analysis was based on the measurements of hematocrit value (Hct), whole blood viscosity (*η*) at four chosen shear rate (*γ*′) values (0.1, 1, 10, and 100 s^−1^), plasma viscosity (*η*_*p*_), complex viscosity (η*), and its elastic (*η*′′) and viscous (*η*′) components measured for four chosen amplitudes (*γ*_0_′) of shear rate values (0.2, 1, 10, and 20 s^−1^). The blood samples were collected into BD Vacutainer Tubes containing lithium heparin (17 international units/mL of blood). The hematocrit value was determined using the standard method. Blood plasma was obtained by centrifugation of whole blood at 4000 rpm for 10 minutes and collection of the supernatant. All viscosity measurements were performed by means of a rotary-oscillating rheometer, Contraves LS40, at the temperature of 37°C. The measurement cell consisted of a steel cup driven by a precise step motor and a steel bob suspended on a quartz thread, which together formed a Couette cell with a volume of 1.8 mL. In the rotary measurements the motion of the cup was unidirectional, and the torsion angle of the thread was taken as the measure of shear stress. In the oscillatory ones the cup was moved in an oscillating way with a constant frequency and both the amplitude of the thread torsion and the phase difference of the cup and bob oscillations were used to calculate the real and imaginary components of the complex viscosity.

Whole blood viscosity was measured in the order of decreasing shear rate in the range 100–0.01 s^−1^ within 5 minutes after short preshearing at *γ*′ = 100 s^−1^ [15, 16]. Plasma viscosity was estimated from a regression analysis of shear rate dependence of shear stress measured in the range of *γ*′ = 10–100 s^−1^. Oscillatory measurements of the viscous and elastic parts of the complex blood viscosity were performed at a constant frequency of 0.5 Hz and decreasing amplitude [9]. Conclusions with regard to the red cells aggregability and deformability were drawn indirectly from the analysis of the flow curves using the rheological model of Quemada ((2) and (3)) and comparing the values of the model parameters: *k*_0_, *k*_*∞*_, and *γ*_*c*_′. This model has been formulated for a suspension of aggregating particles—a substance resembling the properties of real whole blood. The parameters *k*_*∞*_ and *k*_0_ in (2) have the meaning of a measure of red cells rigidity and aggregability, respectively. Parameter *k*_*Q*_ has the sense of erythrocyte intrinsic viscosity which also takes into account the change of erythrocyte shape *via* the function *k*_*Q*_(*γ*′) in (3). The symbols *τ*(*γ*′),  *η*_*p*_, and Hct denote shear stress, plasma viscosity, and hematocrit value, respectively:

(2) $$\begin{array}{c} k_{Q}=\frac{k_{0}+k_{\infty }{\left(\gamma^{\prime }/\gamma_{c}^{\prime }\right)}^{1/2}}{1+{\left(\gamma^{\prime }/\gamma_{c}^{\prime }\right)}^{1/2}}, \end{array}$$

(3) $$\begin{array}{c} \tau \left(\gamma^{\prime }\right)=\eta_{p}{\left[1-\frac{1}{2}k_{Q}\cdot \text{Hct}\right]}^{-2}\cdot \gamma^{\prime }. \end{array}$$

The values of *k*_0_, *k*_*∞*_, and *γ*_*c*_′ were determined from the least squares fit of the model to the experimental flow curve using Hct and *η*_*p*_ as fixed parameters taken from direct measurements.

As a source of the alternating magnetic field the Viofor JPS device (produced by Med & Life Komorów, Poland) was used. The generator was connected to the small applicator—a pad, containing one pair of coils. The peak value of the variable magnetic field of extremely low frequency (ELF) range equaled 56 *μ*T with the mean value of 5 *μ*T. The M1P2 program was used, because in [17] it was found to be the most effective in studies *in vivo*. M1 means that the chosen strength of the magnetic field was constant during the whole application time. The fundamental frequency of pulses was 180–195 Hz. The pulses were administered in the form of packets of pulses (12.5–29 Hz), groups of packages (2.8–7.6 Hz), and series (0.08–0.3 Hz). The base form of impulses was close to the peak shaped one, with a gradual increase in field and a sharp decrease to zero. The changes of polarization took place in the cycle 3-3-2-2 minutes for M1P2 program. Other technical details can be found in the instrument user manual available at the producer web site (currently http://www.medandlife.com/).

## 3. Results

 The results of the rotary measurements, whole blood viscosity values measured for four chosen shear rates and plasma viscosity, are presented in Table 1. For each blood sample the hematocrit value was measured. Hematocrit values remained in the limits from 38 to 51% and its mean value in the group was (44.0 ± 0.5)%.

**Figure 1:**
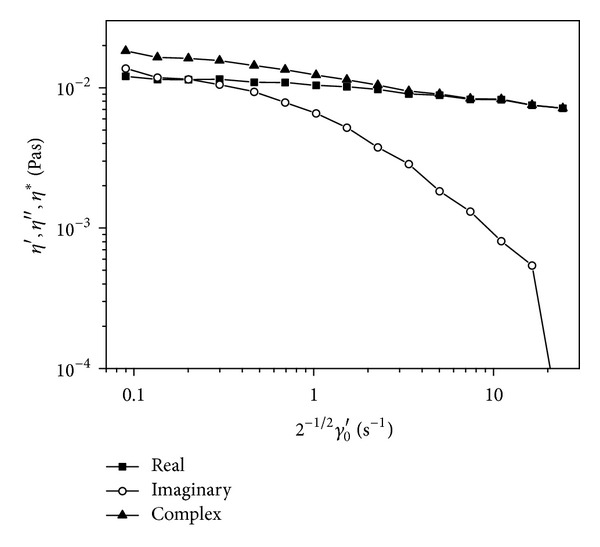
Dependence of the viscous and elastic components of the complex viscosity and the complex viscosity on the rms value of the shear rate amplitude *γ*_0_′ (for oscillation frequency of 0.5 Hz) in the studied group of healthy patients. The rms value of a sinusoidal function is by factor 2^1/2^ smaller than the amplitude.

 The Quemada rheological model parameters values *k*_0_, *k*_*∞*_, and *γ*_*c*_′ and the values of other rheological parameters—plasma viscosity and blood viscosity for four chosen shear rates—are listed in Table 1. The average values of the viscous (*η*′) and elastic (*η*′′) components of the complex viscosity (η*) for four chosen shear rate amplitudes  *γ*_0_′ are also presented in Table 1. In Figure 1 we showed a typical dependence of  *η*′, *η*′′, and η* on shear rate amplitude *γ*_0_′ obtained with oscillations of constant frequency of 0.5 Hz and variable amplitude for one of the samples. 

**Figure 2:**
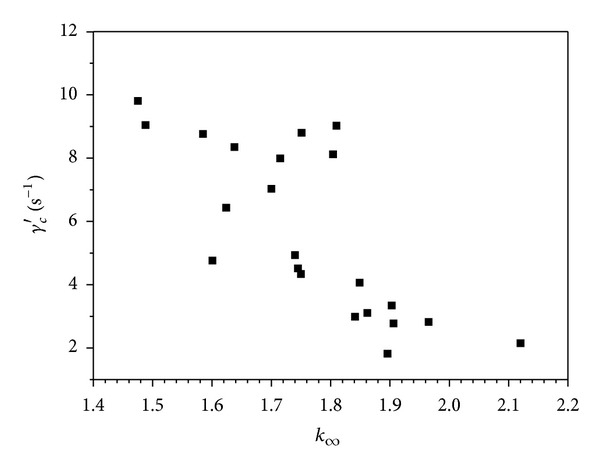
Correlation between the parameters of Quemada model: *γ*_*c*_′ and *k*_*∞*_.

**Figure 3:**
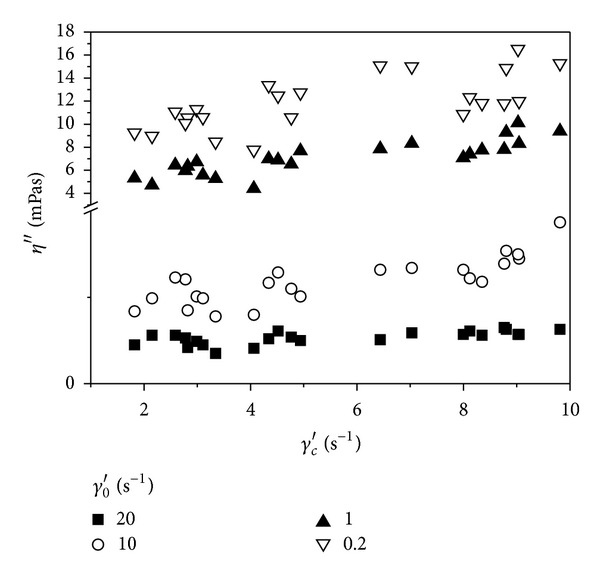
Correlation between the elastic component of the complex viscosity and the *γ*_*c*_′ parameter of Quemada model.

**Figure 4:**
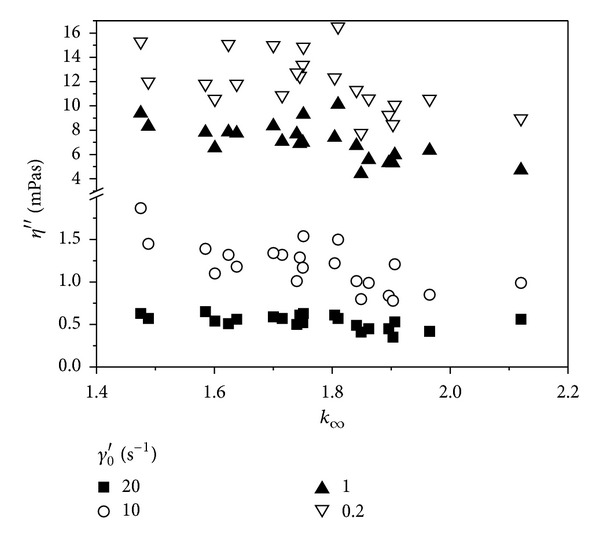
Correlation between the elastic component of the complex viscosity and the *k*_*∞*_ parameter of Quemada model.

In Figure 2 we showed correlations between a pair of Quemada model parameters representing red cells rigidity and deformability  *γ*_*c*_′ and  *k*_*∞*_. Correlations between Quemada model parameters  *γ*_*c*_′ and *k*_*∞*_ and the elastic component  *η*′′ of the complex viscosity are presented in Figures 3 and 4.

## 4. Discussion

In this work we have studied the effect of low frequency magnetic field on the rheological properties of blood. This issue has not been analyzed in detail so far. Our studies have been performed *in vitro*, with regard to both magnetic field application and blood viscosity measurements. We analyzed rheological properties of blood collected from healthy patients, obtained by means of a rotary-oscillating rheometer, Contraves LS40. Although it is possible to perform application of MF *in vivo*, as it was shown in [17], measurements of blood viscosity have to be performed *in vitro*. There are some methods to measure blood flow through certain vessels *in vivo* but they do not provide information about blood viscosity alone. 

Hemorheological studies are based on the evaluation of blood fluidity *in vitro* in conditions mimicking the physiological ones and on investigating the relations among individual physical parameters of blood components. Parameters of special interest are red cells deformability and ability to orient in flow, their aggregability, whole blood viscosity, blood plasma viscosity, and complex blood viscosity. 

In our current *in vitro* studies only weak effects of magnetic field therapy on blood rheological parameters are observed. Statistical analysis of observed differences showed their statistical insignificance. However, the results obtained with different methods were consistent. Moreover, differences recently found in a recent *in vivo* study [18] may simply reflect the influence of magnetic field on the whole organism and not only the blood itself.

The values of relative blood viscosity for four chosen shear rates, measured in the rotary experiment, are listed in Table 1. These results are comparable with the data published in the literature obtained from control group in other hemorheological studies [19]. Observed differences are of no statistical significance (*P* < 0.06), and even if treated seriously, they could result from the fact that the control groups in the cited works were composed of patients free from circulatory system disorders; however they were hospitalized and hence not healthy. In the current study all blood donors were healthy. The values of relative blood viscosity measured in this study are comparable to results obtained in the literature using different methods and for different groups of healthy patients [7, 8, 20, 21]. No statistically significant differences have been found between blood samples before and after administration of the magnetic field; however, there seems to be a tendency of decreased blood viscosity at low shear rates (Table 1,  *γ*′ = 0.1 s^−1^).

 Plasma viscosity measurements were performed by means of the same rotary-oscillating rheometer but using only rotary techniques. In this study the plasma composition was not measured and hence no correlations between the plasma protein composition and plasma viscosity could be found. Plasma protein composition is a very important parameter in the analysis of plasma viscosity in the case of patients with diagnosed circular system disorders [22]. Analysis of the magnetic field effect on blood plasma viscosity revealed statistically significant differences between the plasma samples before and after administration of magnetic field to the blood samples (Table 1). Increased plasma viscosity might result from a change of plasma proteins conformation but this hypothesis was not verified in this study. The values of plasma viscosity for healthy people obtained in this study are comparable to the results reported in the literature for similar studies of control groups [19].

Mathematical analysis of the whole blood flow curves obtained from rotary measurements with the use of Quemada rheological model allows for overcoming this problem and obtaining reliable information about red cells aggregability and deformability from Quemada model parameters (Table 1). The values of Quemada model parameters, *k*_0_, *k*_*∞*_, and  *γ*_*c*_′, are comparable to those obtained in the literature [12, 19, 20]. Analysis of the correlation among different model parameters revealed a negative correlation between the  *γ*_*c*_′  parameter indicating the moment of erythrocytes beginning to form packages (*rouleaux* formation) and the parameter *k*_*∞*_ interpreted as a measure of red cells rigidity (Figure 2). These two parameters reflect two different phenomena. The fact of correlation between them means that the red cells rigidity and the moment of rouleaux formation are interdependent either directly or through another parameter/physical phenomenon.

Ciejka and Gorąca [23] studied the effect of magnetic fields with parameters used commonly in magnetotherapy on chosen biochemical parameters of rats blood. They found that variable magnetic field of low frequency administered daily for 30 min induces in the animals changes of morphological parameters of blood (decrease in red cells number after 14 days followed by an increase after 28 days and an increase in hemoglobin level after 28 days) and disorders in water-electrolyte balance. These changes vanish after completing the therapy.

Cakir et al. [24] studied the effect of 50- and 100-day application of low frequency magnetic field of 0.97 mT amplitude for 3 hours per day on the hematological rats blood parameters. They found that magnetic fields from the ELF range induce small but statistically significant changes remaining in physiological range of some rats blood parameters, namely, a small decrease in eosinophil, hemoglobin, and mean platelet volume (MPV) levels. 

In this study we have analyzed the effect of magnetic field not only on whole blood and blood plasma viscosity but also on the viscoelastic properties of blood. The complex viscosity of blood was measured at variable shear amplitudes and constant oscillation frequency of 0.5 Hz. Such a choice of the experimental method was dictated by the technical limitations of the experimental setup. In the case of other oscillation frequencies or shear amplitude ranges the mechanical resonance effects strongly affected the measurement [9]. The measurement of the viscous (*η*′) and elastic (*η*′′) components of the complex blood viscosity (η*) allows for studying the viscoelastic properties of blood which also reveals information about red cells aggregability and deformability. These two features strongly affect blood flow in the circulatory system, especially in microcirculation [18, 25, 26].

Viscoelastic properties of blood change as a function of shear rate which is reflected by the results listed in Table 2. For small values of shear rate amplitude  *γ*_0_′ = 0.2 we found statistically significant lowering of the viscous (*η*′) component of the complex viscosity. Complex viscosities measured by rotary-oscillating rheometer shown in Figure 1 are comparable to those obtained with an oscillating (1 Hz) capillary rheometer [26, 27]. The value of the elastic component *η*′ refers mainly to the packages of red cells which are broken by increasing value of the shear amplitude  *γ*_0_′. It can be seen that starting from a certain value of  *γ*_0_′ only individual red cells contribute to the elastic component of red cells complex viscosity [26]. Since individual red cells are rather soft, in the range of high shear rates practically only viscous properties of blood are observed.

In the literature it has been described that there is a connection between the red cells sedimentation rate and the values of the viscous (*η*′) and elastic (*η*′′) components of the complex blood viscosity (η*) [18]. In the current study the sedimentation rate was not measured on the day of blood uptake for rheological measurements. Performing sedimentation experiments would allow for such comparison and would provide another source of information on red cells aggregability. However, even the fact that low values of the viscous component alone in the range of low shear rates (*γ*_0_′ → 0) are accompanied by high value of  *η*′ for high shear rates (*γ* → *∞*) may be an indication of faster red cells aggregability [18].

The values of the elastic (*η*′′) component of the complex blood viscosity were correlated with the  *γ*_*c*_′ and  *k*_*∞*_ parameters of Quemada rheological model. The former parameter indicates the moment of rouleaux formation, while the latter is interpreted as red cells rigidity (Figures 3 and 4). In the range of low shear rate amplitudes  *γ*_0_′ a slight positive correlation between the  *η*′′ component and the  *γ*_*c*_′ parameter can be seen and a negative one between  *η*′′ and  *k*_*∞*_ can be found. For the shear rate amplitude of 20 s^−1^ no correlations were found.

 Comparing the values of  *η*′ and  *η*′′ obtained in this study (Table 1) with analogous data found in a group of patients after myocardial infarction [9], complex viscosity components are higher in the group of healthy patients compared to the patients after myocardial infarction. Observed differences were statistically significant (0.001 < *P* < 0.004). A comparative analysis of these results is possible because both studies were performed by means of the same instrument and using the same method. Such a result may be interpreted as a result of the influence of the therapy applied to the patients after myocardial infarction on the viscoelastic properties of blood. In this study no comparison with other control groups has been performed.

It is also interesting to compare this *in vitro* study to some recent results of *in vivo* magnetotherapy effects on blood viscosity in a group of patients with cerebrovascular disease [17], where a tendency to decreasing red cells aggregability was found after magnetic field application. Such result was interpreted in terms of manifestation of some autoregulatory mechanisms in living organisms induced by variable magnetic field therapy, as it was suggested in other studies [28, 29]. It is obvious that in the case of the *in vitro* studies such mechanisms can not be observed.

*In vitro* measurements of blood sedimentation rate in the presence of magnetic field should be possible with a small modification of the setup. It could be good complementary information useful in the interpretation of rheological data.

## 5. Conclusions

 In this study a comprehensive analysis of rheological properties of blood taken from a group of healthy patients, qualified as blood donors, was performed. On the basis of the measurements of whole blood, plasma, and complex viscosities by means of a rotary-oscillating rheometer in a group of healthy patients, the following conclusions can be drawn.

1. In the *in vitro* conditions only weak effects of magnetic field therapy on blood rheological parameters are observed. Statistical analysis of observed differences showed their statistical insignificance.
2. Comparison of these results with studies performed *in vivo* suggests that the lack of effect of magnetic field application *in vitro* might result from the fact that magnetotherapy influences the whole organism and not only blood.
3. The results of rotary measurements obtained in this study remain in agreement with similar studies reported in the literature for healthy people.
4. The results of oscillatory measurements are in agreement with the flow curve measurements.
5. The results of the oscillatory measurements are in agreement with the rheological model of Quemada.
6. Due to technical limitations, the viscoelastic properties of the system could only be measured using the method of constant frequency and variable shear amplitude which couldnot exceed 20 s^−1^.
7. In the case of oscillatory measurements, sedimentation experiments would be very helpful.

## Figures and Tables

**Table 1:** The values of blood rheological parameters and Quemada's model in the group of healthy patients before and after magnetic field.

Rheological parameter	Group	
	Before magnetic field (*n* = 25)	After magnetic field (*n* = 25)
Hematocrit (Hct)	44.0 ± 0.5	44.0 ± 0.5
Plasma viscosity (*η*_*p*_) [mPas]	1.33 ± 0.03*	1.41 ± 0.01
Relative blood viscosity at 0.1 s^−1^	39.04 ± 2.47	36.88 ± 2.18
Relative blood viscosity at 1 s^−1^	15.84 ± 0.79	15.29 ± 0.75
Relative blood viscosity at 10 s^−1^	6.47 ± 0.24	6.35 ± 0.22
Relative blood viscosity at 100 s^−1^	3.62 ± 0.09	3.54 ± 0.08
Quemada model parameter *k*_0_	4.16 ± 0.07	4.15 ± 0.06
Quemada model parameter *k*_*∞*_	1.77 ± 0.04	1.94 ± 0.23
Quemada model parameter *γ*_*c*_′	5.78 ± 0.66	6.08 ± 0.64

*P < 0.02.

**Table 2:** The values of the viscous and elastic components of the complex viscosity in the group of healthy patients before and after magnetic field.

Rheological parameter	Before magnetic field (*n* = 25)	After magnetic field (*n* = 25)	*P*
Value of *η*′ at γ_0_′ = 0.2 s^−1^	9.25 ± 0.44	8.08 ± 0.33	<0.04
Value of *η*′ at γ_0_′ = 1 s^−1^	10.76 ± 0.36	10.85 ± 0.37	—
Value of *η*′ at γ_0_′ = 10 s^−1^	8.70 ± 0.21	8.78 ± 0.26	—
Value of *η*′ at γ_0_′ = 20 s^−1^	7.92 ± 0.19	8.03 ± 0.23	—
Value of *η*′′ at γ_0_′ = 0.2 s^−1^	12.03 ± 0.46	12.15 ± 0.56	—
Value of *η*′′ at γ_0_′ = 1 s^−1^	7.20 ± 0.31	7.40 ± 0.39	—
Value of *η*′′ at γ_0_′ = 10 s^−1^	1.22 ± 0.06	1.22 ± 0.06	—
Value of *η*′′ at γ_0_′ = 20 s^−1^	0.54 ± 0.02	0.55 ± 0.02	—
